# Global avian influenza outbreaks 2010–2016: a systematic review of their distribution, avian species and virus subtype

**DOI:** 10.1186/s13643-018-0691-z

**Published:** 2018-01-25

**Authors:** Ioanna P. Chatziprodromidou, Malamatenia Arvanitidou, Javier Guitian, Thomas Apostolou, George Vantarakis, Apostolos Vantarakis

**Affiliations:** 1 Department of Public Health, School of Medicine, University of Patras, Patras, Greece; 2 Department of Hygiene and Epidemiology, School of Medicine, University of Thessaloniki, Thessaloniki, Greece; 3 Royal Veterinary College, London, UK; 4 Department of Physiotherapy, School of Health Professionals, Alexander Technological Educational Institute of Thessaloniki, Thessaloniki, Greece; 5 Prefecture of Western Greece, Patras, Greece

**Keywords:** Avian influenza, Avian flu, Outbreak, Avian species, Wild birds

## Abstract

**Background:**

We conducted a systematic review to investigate avian influenza outbreaks and to explore their distribution, upon avian influenza subtype, country, avian species and other relating details as no comprehensive epidemiological analysis of global avian influenza outbreaks from 2010 to 2016 exists.

**Methods:**

Data was collated from four databases (Scopus, Web of Science Core Correlation, PubMed and SpringerLink electronic journal) and a global electronic reporting system (ProMED mail), using PRISMA and ORION systematic approaches. One hundred seventy three avian influenza virus outbreaks were identified and included in this review, alongside 198 ProMED mail reports.

**Results:**

Our research identified that the majority of the reported outbreaks occurred in 2016 (22.2%). These outbreaks were located in China (13.6%) and referred to commercial poultry farms (56.1%). The most common subtype reported in these outbreaks was H5N1 (38.2%), while almost 82.5% of the subtypes were highly pathogenic avian influenza viruses. There were differences noticed between ProMED mail and the scientific literature screened.

**Conclusions:**

Avian influenza virus has been proved to be able to contaminate all types of avian species, including commercial poultry farms, wild birds, backyard domestic animals, live poultry, game birds and mixed poultry. The study focused on wet markets, slaughterhouses, wild habitats, zoos and natural parks, in both developed and developing countries. The impact of avian influenza virus seems disproportionate and could potentially burden the already existing disparities in the public health domain. Therefore, a collaboration between all the involved health sectors is considered to be more than necessary.

**Electronic supplementary material:**

The online version of this article (10.1186/s13643-018-0691-z) contains supplementary material, which is available to authorized users.

## Background

Avian influenza virus (AIV) can cause severe outbreaks in the poultry population. Nevertheless, it may occasionally infect humans exposed to infected poultry. The term outbreak refers to a number of cases of a specific disease in excess of normal endemicity. However, an outbreak is not always defined by a specific number of cases. The relative occurrence varies upon the infectious agent, the composition of the population exposed and prior exposure or lack of exposure of the population to the certain infectious agent (immune system status). Moreover, the time and the place of occurrence also play a significant role in identifying an outbreak. Therefore, an outbreak refers to a specific population (community), at a specific time point (season) and in a specific place (geographic area) [1–4]. On the other hand, a case is considered as the occurrence of a single individual (i.e. wild bird) with a disease, and its detection is of great importance as an outbreak typically depends on the detection of individual cases [5].

Avian influenza is an A-type influenza virus and belongs to the *Orthomyxoviridae* family. Type-A influenza theoretically contains thousands of different antigenic subtypes, due to the combination between the main virion antigens, haemagglutinin (HA) and neuraminidase (NA) [6]. Until recently, 16 HA subtypes and 9 NA subtypes have been recognised, while 2 additional HA and NA subtypes have been identified in bats [7, 8]. The term “highly pathogenic avian influenza” (HPAI) generally refers to the strains that may induce “intravenous pathogenicity index” (IVPI) greater than 1.2 or mortality rate over 75% in a defined chicken population during the specified interval of 10 days. Using this definition, all the HPAI strains isolated to date are of H5 and H7 subtype. However, viruses of these subtypes can also be of low pathogenicity.

According to the World Organisation for Animal Health (OIE), AIV is defined as “an infection of poultry caused by any influenza A virus with high pathogenicity (HPAI) and by H5 and H7 subtypes with low pathogenicity (H5/H7 LPAI)” [9]. Moreover, OIE requires notification for all H7 and H5 subtypes, regardless of their pathogenicity, as they have the potential to mutate into HPAI viruses [10]. In other words, non-H5 and non-H7 low pathogenic avian influenza (LPAI) are not determined as notifiable.

The symptoms of AIV infection vary widely, depending on the infected species, the age, the sex, the strain, the subtype involved, concurrent infections and, of course, many predictable and non-predictable environmental factors. The clinical manifestations vary from mild to severe respiratory, nervous, reproductive and gastrointestinal system disorders. There are also cases of no clinical signs and sudden deaths [11].

Generally, the economic consequences of AIV in poultry are severe, since not only the production of eggs is affected, but also the quality of the egg influenced. In addition, a high mortality in birds is observed, as well. Moreover, given that AIV determines, to a certain extent via genetic reassortment [12], the development of advanced human strains, AIV is a major subject to be taken into account as far as public health is concerned [13].

The year 2010 was the fifth year when measures and surveillance programmes were imposed by proper directives, and legislation was enforced by all proper authorities. Therefore, 2010 is considered as the time when all procedures were routinely established. Comprehensive epidemiological analysis of global avian cases of AIV is scarce, and a few studies have presented in detail the changing epidemiology of AIV cases [14–16]. To improve the understanding of AIV epidemiology, we conducted a systematic review of the AIV outbreaks to describe the distribution and the magnitude of all avian cases which occurred globally during the period 2010–2016.

## Methods

An a priori protocol was performed, based on the PRISMA statement [17], to specify the search strategy, the eligibility criteria, the objectives and the methods of this systematic review. However, this systematic review was not registered with PROSPERO (International prospective register of systematic reviews).

### Eligibility criteria

All study design types were included except experimental studies, since they are regarded as artificially induced cases rather than naturally occurring cases. All AIV outbreaks dated from 2010 to 2016 were investigated. Papers that did not clarify the exact number of cases (birds, not flocks) in each outbreak, during the period of interest, were approached through the ProMED mail tool to seek additional information. If no extra information was found, they were excluded. No publication status restrictions were imposed. In terms of methodology, we should also point out that the “scale” at which an outbreak is defined, especially for domestic poultry, could be defined from one bird in some reports to one farm in others. All laboratory-confirmed cases or outbreaks where confirmation was performed by officially indicated methods (serological, molecular, both serological and molecular, generally characterised advanced laboratory method or specific pathogen-free embryonate eggs) were included [18]. All AIV subtypes and both HPAI and LPAI were included. All avian species of any age were considered, including wild birds; commercial poultry; backyard domestic poultry; wet market, slaughterhouses, wild habitats, zoos, natural park and village species; game birds and live bird and poultry market species. Although some of these species are already covered by the above-mentioned groups, we concluded using these terms exactly as identified in the literature screened and included in this systematic review. In silico and in ovo studies (943), socio-financial studies (899), studies with phylogenetic and evolutionary patterns of various AIV isolates (1356), studies concerning human AIV cases (401) and, generally, papers not stating clearly and specifically the number of cases and types of AIV isolated (13) and for mixed other reasons (1063) were not included in this study, as they did not contain specific outcomes relevant to our systematic review. No limits regarding language were applied. All non-English language papers (including French, Spanish, German, Chinese, Japanese and others) were translated by Google translator and were, thus, included in this systematic review. Hand searching authors’, experts’ or manufacturers’ opinions; conference proceedings; editorials and letters to the editors were not included in our study.

The selection criteria developed a priori were the following:

- Number of cases of the outbreak
- Year of the outbreak
- Country/city of epidemic
- Surveillance/vaccination programme administration
- Age group of cases
- Epidemiological unit of epidemic (commercial poultry, backyard domestic poultry, etc.)
- Type of samples (blood, swab, etc.)
- Method of analysis (serological, molecular, etc.)
- Symptoms
- Vicinity of water
- Low/highly pathogenic avian influenza subtype
- AIV subtype

### Information sources and search strategy

The search and selection study and analysis process was based on PRISMA and ORION statements’ guidelines [19, 20]. Data search was run from December 1, 2015 to December 31, 2016, in an attempt to scrutinise all the available literature. Peer-reviewed articles concerning AIV were identified through an assiduous search applied to various electronic databases such as Scopus, Web of Science Core Correlation, PubMed and SpringerLink electronic journal. Grey literature was, also, investigated via the global electronic reporting system Program for Monitoring Emerging Diseases (ProMED mail). ProMED mail, which collaborates with the International Society of Infectious Diseases, is, among others, supported by OIE. Thus, OIE was also, indirectly, used as a data source. In addition, references of articles obtained by electronic databases which were considered to be related to our study were acquired by Swetswise, a portfolio of library-oriented tool (containing books, journals and databases). Scopus was selected to be our starting point of research.

The search terms used to investigate the reported databases were “avian influenza AND outbreak”, “avian influenza AND cases”, “avian influenza AND case”, “avian flu AND case” and “avian flu AND cases”, published from 2010 to 2016. The search queries were set to include the above terms in (a) article titles, abstract and keywords as for Scopus database, (b) topic (kw) and title as for Web of Science Core Correlation and (c) all fields as for PubMed/MEDLINE and SpringerLink.

A full electronic search strategy utilising the Web of Science Core Correlation database is presented in Table 1.

**Table 1:** Search strategy (Web of Science Core Correlation), conducted in January 2017

1.	“avian influenza AND case”.mp
2.	“avian influenza virus AND case”.mp
3.	“avian flu AND case”.mp
4.	1 or 2 or 3
5.	“avian influenza AND outbreak”.mp
6.	“avian influenza virus AND outbreak”.mp
7.	“avian flu AND outbreak”.mp
8.	5 or 6 or 7
9.	limit 1 or 2 or 3 or 5 or 6 or 7 to publication years =“2010-2016”
10.	limit to document types=“articles” and “reviews”

### Study selection

An eligibility assessment process was performed, independently, in a standardised manner, mainly by two reviewers (I. P. Chatziprodromidou, A. Vantarakis), in an attempt to evaluate and confirm relevant data to the topic reviewed. Disagreements were resolved by a discussion among all authors, leading to a final consensus.

### Data collection process and items

A data extraction sheet was developed, based, mainly, on the rationale proposed by the Cochrane Consumers and Communication Review Group [21]. Initially, this was pilot-tested in the first 20 articles selected to be included in our study, and it was refined according to the needs that emerged. The first author (IPC) was responsible for the data extraction from the studies selected to meet the eligibility criteria set, and the last author (AV) was responsible for re-evaluating and confirming the research findings of the first author. Duplicate publications were identified and taken into account only once, with the aid of Mendeley, a free reference manager and research paper organiser. No final disagreements between the authors ever remained.

The information extracted from the articles selected are summarised in Table 2.

**Table 2:** Data sheet extracted from articles and records included in this systematic review

Data	Range
Year published	2010–2016
Number of samples taken/susceptible	0–1,904,500
Number of cases of AIV	1–587,160
Surveillance program	Yes/no
Vaccination program	Yes/no
Age of animals (days)	20–420
Epidemiological unit	Wild bird species
	Commercial poultry
	Backyard domestic poultry
	Live bird market/live poultry market
	Wet market
	Slaughterhouse
	Wild habitat
	Zoo
	Natural park
	Village
	Mixed
Type of samples	Blood
	Swab (cloaca, pharynx, conjunctiva)
	Tissue
	Faeces
	Other
	Mixed
Symptoms	Symptoms of respiratory disease
	General symptoms
	Symptoms of reproductive system
	Dead
	Other
	Not mentioned
Flock size	14–7,498,221
Vicinity of water	Yes/no
Method of testing	Molecular
	Serological
	Both molecular and serological
	Specific-pathogen-free 6 embryonated chicken eggs
	Advanced laboratory testing methods
Low (LPAI) or highly (HPAI) pathogenic avian influenza subtype	LPAI
	HPAI
Haemagglutinin and Neuraminidase type	(More than 75 combinations)

### Assessment of risk of bias in included studies

As with all research studies, the whole format of design and conduct of reviews may introduce biases that may affect the systematic review findings. Two reviewers (IPC, AV), independently and in a blind manner, assessed the quality of the included studies. A third reviewer (MA) also evaluated the studies which were selected to be included in this research, and no studies were excluded. The quality assessment of each included study was based on McMaster Critical Review Form for quantitative studies [22] accompanied by a concrete guideline [23]. All included studies were assessed based on three criteria: (a) sample: this was evaluated in order for the selection bias to be reduced (i.e. if the sample size tested was representative of the studied population), the sample size to be sufficient and according to the characteristics of the participants, (b) measurement: this was assessed relatively to measurement bias being minimised and (c) analyses: these were evaluated concerning the properness of the analysis followed to answer the research question (i.e. statistical significance) [24]. Those three criteria were evaluated and scored as “a”: no criteria were met within this component, “b”: only some of the criteria met within this component, “c”: all criteria were met within this component and “unclear”: no data provided.

## Results

### Search results

During the selection process, Scopus yielded 1712 records, Web of Science Core Collection 1557 records, PubMed 1512 records and SpringerLink 2242 results. All these added to 7023 articles, of which 2198 were duplicates, leaving a total of 4825 distinct reports. ProMED mail had an additional 198 records fitting our research strategy criteria.

### Study selection

**Fig. 1:**
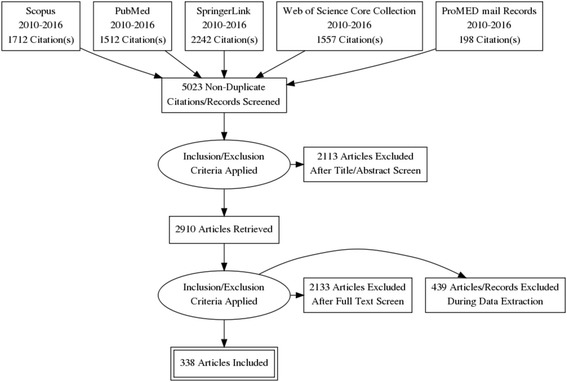
Flow of information through the different phases of this systematic review

The study selection process is summarised in a flow diagram (Fig. 1). As mentioned, the search provided initially 7023 citations. After eliminating duplicates, 5023 remained. After the initial review of all these studies by the first and last author and after subsequent discussion and consensus of the remaining authors, 2113 articles/records were discarded after screening, both the title and the abstract while 2572 articles/records were discarded as they did not meet the eligibility criteria, after a full-text screening or/and during the data extraction process. Three additional studies were screened, which met the eligibility criteria but were discarded as the full texts were not available. A total of 173 publication articles from the electronic databases mentioned, and 198 records from ProMED mail were eventually deemed eligible for inclusion in this systematic review. Unpublished related literature was not included.

### Assessment of risk of bias in included studies

The overall agreement percentage was calculated and considered as substantial for all sample, analyses and measurement criteria. Only 13 of the included studies received the maximum score for all criteria.

### Characteristics of included studies

The greatest number of studies and records (144, 22.2%) occurred in year 2016, while 142 (21.9%) occurred in 2015, 105 (16.2%) in 2014 and 82 (12.6%), 56 (8.6%), 67 (10.3%) and 52 (8%) in 2013, 2012, 2011 and 2010, respectively. Taking into account the outbreaks outsourced by ProMED mail, which were instantly (real-time) announced, most of the records (143, 27.3%) were published in 2016, 134 (25.6%) in 2015, 74 (14.1%) in 2014, 52 (9.9%) in 2013, 33 (6.3%) in 2012, 47 (9%) in 2011 and 39 (7.4%) in 2010.

**Fig. 2:**
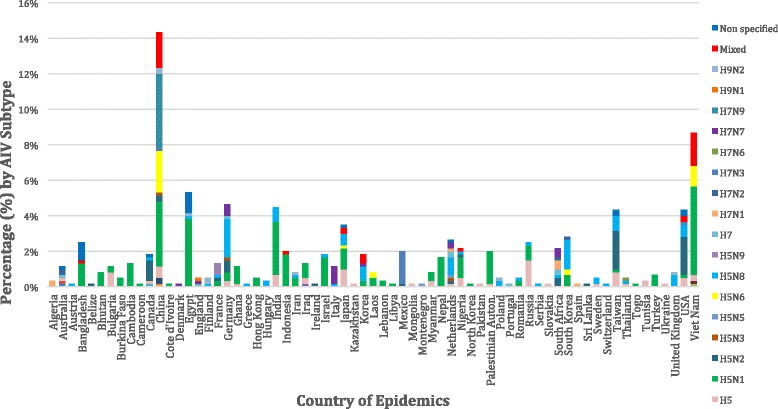
Avian influenza virus subtypes upon country

**Fig. 3:**
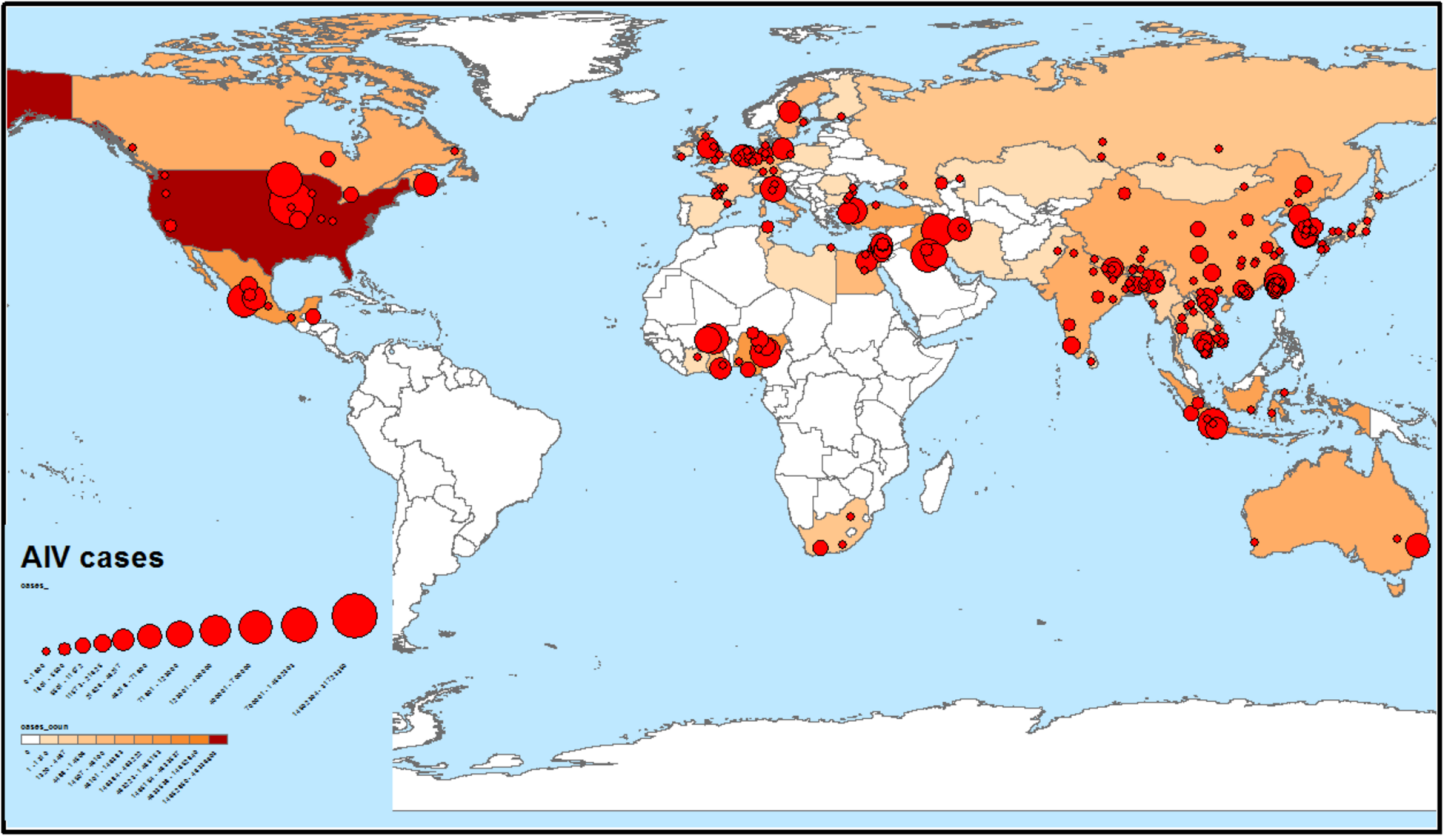
Global distribution of avian influenza virus outbreaks

Regarding the “country of epidemics”, we estimated that the largest number of outbreaks (88, 13.6%) occurred in China, while 53 (8.2%), 32 (4.9%), 29 (4.5%), 27 (4.2%), 25 (3.9%) and 23 (3.5%) occurred in Viet Nam, Egypt and Germany, India and the USA, Taiwan, the Netherlands and Israel, respectively. AIV subtypes recorded per country are presented in Fig. 2. Subsequently, we conducted a world spot map, based on outbreaks recorded in each country during 2010–2016 [25] (Fig. 3). The largest outbreak reported in the scientific literature and ProMED mail took place in Jalisco, Mexico. This outbreak occurred in 2012. During this outbreak, it was estimated that 3,987,160 birds from commercial poultry species were infected by HPAI H7N3.

### AIV cases

The studies and records (ProMED mail) included a total of 58,709,463 individual birds. Most of the outbreaks (364), which are included in this systematic review, originated from commercial poultry farms (56.1%), followed by wild bird species (103 outbreaks, 15.9%), backyard domestic poultry (87 outbreaks, 13.4%), mixed (commercial and wild) species (26 outbreaks, 4%), live poultry market species (17 outbreaks, 2.6%), live bird market species (16 outbreaks, 2.5%), village species (11 outbreaks, 1.7%), natural park species (7 outbreaks, 1.1%), wet market species (5 outbreaks, 0.8%), zoo species (4 outbreaks, 0.6%), wild habitat species (3 outbreaks, 0.5%), slaughterhouse species (1 outbreak, 0.2%) and finally, game birds (1 outbreak, 0.2%).

The flock size ranged between 14 and 7,498,221 but was not available in almost half (292 out of 649) of the outbreaks included. In 44.99% of the outbreaks, the flock size was not reported either because there was no need as the data was referring to wild species or because this information was indeed missing.

In only 138 outbreaks (21.3%), a vaccination programme was mentioned; however, no access to more precise information was feasible concerning the subtypes covered, the doses received, etc. Regarding the administration of the surveillance programme at the time of the outbreak included, only in 252 outbreaks (38.8%), which were assessed, it was reported that a surveillance programme was implemented, without precisely mentioning the syllabus and the format of the programme (age, species, subtypes covered, etc.).

The bird age was between 20 and 420 days; however, the factor “age” was mentioned only in 5.39% of the studies and records (35) included; 94.6% of the outbreaks failed to outline the age, and for this reason, the value age could not be reliably evaluated. Among those mentioning the specific case age, 61.8% were aged between 0 and 100 days old, 29.4% between 101 and 200 days old and 2.9% between 301 and 400, 201 and 300 and > 401 days old.

The symptoms of AIV were not mentioned in 58.4% of the outbreaks, while 16.9% outlined mixed symptoms (a drop in feed and water intake, a drop in egg production, lack of vocalisation, depression, mortality, coughing, disability in breathing, respiratory signs, fever), 15.7% of the cases mentioned mortality of the birds, 2.3% of the cases referred symptoms of the respiratory system, 2.2% of the cases cited other symptoms and 0.2% of the cases pointed out symptoms of the reproductive system and general symptoms.

The sample type tested was missing in almost 63% of the outbreaks. Whenever stated, 36.7% were characterised as mixed (blood, oropharyngeal, conjunctiva, pharynx and faecal swabs, tissue, faeces, carcass), 35.4% of the samples were stated as “other” (like feathers, due to feather tropism of specific AIV subtypes noted in experimentally infected avian species) [26], 20.4% were swabs (cloaca, pharynx and conjunctiva), 3.8% blood samples, 2.5% faeces and 1.3% specific tissue.

Information regarding “vicinity to water” in relation to the outbreak area was missing in 75.5% of the outbreaks. However, whenever vicinity to water was mentioned, water was present in 97.5% of the cases; vicinity to water is considered as a significant risk factor because it is regarded as a wild species habitat for most of the cases [27]. The subtypes reported in outbreaks, where vicinity of water was mentioned, were H5N1 (43 outbreaks, 28.5%), H5N8 (34 outbreaks, 22.5%), mixed (24 outbreaks,15.9%), H5 (18 outbreaks, 11.9%), H5N6 (9 outbreaks, 6%), non-specified subtype (5 outbreaks, 3.3%), H7N9 (5 outbreaks, 3.3%), H3N8 (2 outbreaks, 1.3%), H10N7 (1 outbreak, 0.7%), H5N9 (1 outbreak, 0.7%), H11N9 (1 outbreak, 0.7%), H1N2 (1 outbreak, 0.7%), H7N2 (1 outbreak, 0.7%), H7N7 (1 outbreak, 0.7%), H4N6 (1 outbreak, 0.7%), H9N2 (1 outbreak, 0.7%) and H5N2 (1 outbreak, 0.7%).

Almost all (97.68%) scientific articles and records found in ProMED mail mentioned the type of testing employed to document AIV infection; 62.6% of the samples were identified by advanced laboratory testing [namely real-time polymerase chain reaction (real-time PCR), real-time reverse transcriptase/polymerase chain reaction (RRT-PCR), virus isolation, virus sequencing], 31.1% were identified by molecular methods, 3.5% of samples were tested with both serological and molecular methods (in most cases, screening was prepared with serological methods and verification for positive and suspect samples was confirmed with molecular methods), 2.5% were tested with serological methods only (enzyme-linked immuno-sorbent assay, Immunoblot) and 0.3% with specific pathogen-free embryonate eggs.

**Fig. 4:**
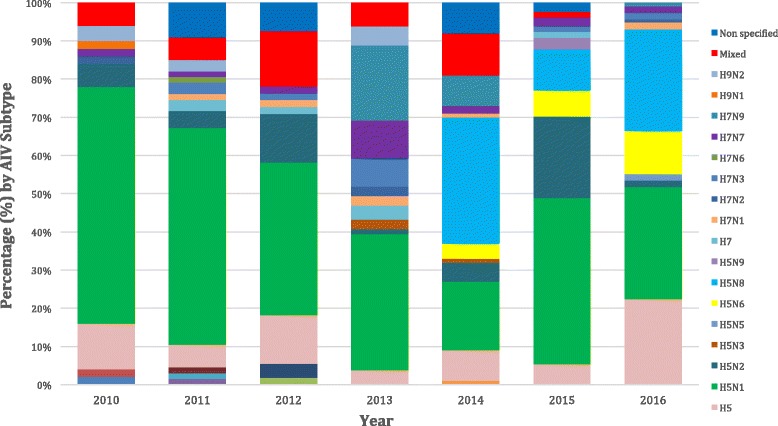
Avian influenza virus subtypes upon year (2010–2016)

**Fig. 5:**
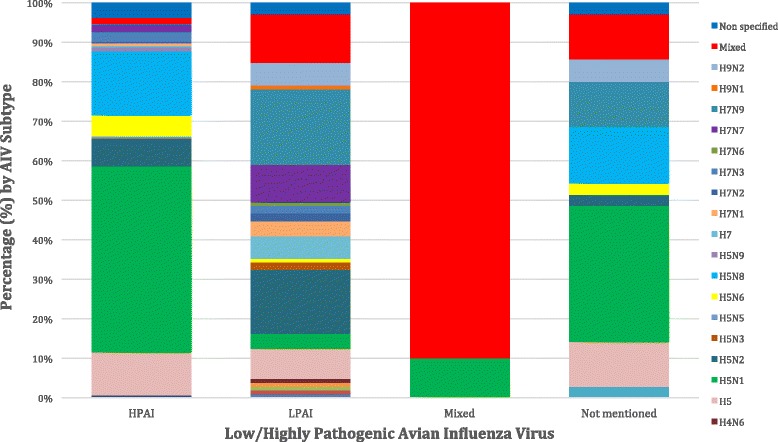
Low(LPAI)/\highly (HPAI) pathogenic avian influenza virus upon avian influenza virus subtype

**Fig. 6:**
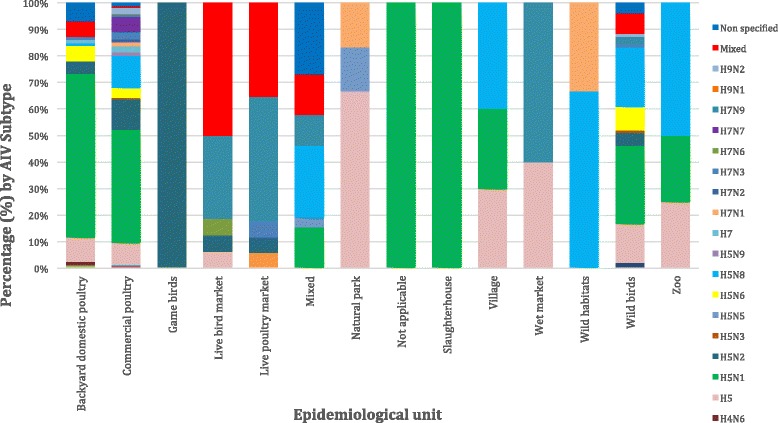
Avian influenza virus subtype distribution upon avian species

Only 3.5% of the included articles did not specify the AIV subtype isolated. AIV subtypes investigation during 2010 to 2016 is clearly demonstrated in Fig. 4, where the presence of H5N1, H5N2 and H5 and other subtypes remains constant and strong. The most often (229 outbreaks, 38.2%) isolated AIV subtype was H5N1, followed by H5N8 (78 outbreaks, 13%), H5 (61 outbreaks, 10.2%), H5N2 (49 outbreaks, 8.2%), mixed subtypes (33 outbreaks, 5.5%), H5N6 (26 outbreaks, 4.3%), H7N9 (25 outbreaks, 3.9%), H7N7 (18 outbreaks, 3%), H7N3 (13 outbreaks, 2.2%), H9N2 (8 outbreaks, 1.3%), H7 (8 outbreaks, 1.3%), H7N1 (7 outbreaks, 1.2%), H7N2 (4 outbreaks, 0.7%), H5N9 (4 outbreaks, 0.7%), H5N3 (3 outbreaks, 0.5%), H3N8 (2 outbreaks, 0.3%), H5N5 (2 outbreaks, 0.3%), H3N2 (1 outbreak, 0.2%), H10 (1 outbreak, 0.2%), H1N2 (1 outbreak, 0.2%), H7N6 (1 outbreak, 0.2%), H4N6 (1 outbreak, 0.2%), H10N7 (1 outbreak, 0.2%),, H9N1 (1 outbreak, 0.2%), H1N1 (1 outbreak, 0.2%) and H11N9 (1 outbreak, 0.2%). The distribution between patterns of HPAI and LPAI infection in bird populations is summarised in Fig. 5. In almost 82.5% of wild bird outbreaks HPAI, was recorded, followed by 9.7% of LPAI, 6.8% not mentioned and 1% mixed outbreaks. Concerning commercial poultry farms, 73.1% were HPAI, 21.7% LPAI and 5.2% were not mentioned. A more detailed case and quantitative approach, namely the precise number of cases of each AIV subtype upon the country, epidemiological unit and year is summarised in (see Additional file 1: Table S1). AIV subtype distribution upon avian species is summarised in Fig. 6, where commercial poultry seems to be “hit” more than any other species and mostly by H5N1 subtype, while wild birds were, also, mostly “hit” by H5N1, but also by various other AIV subtypes. Notably, H5N8 subtype entered Europe with its first appearance in Germany.

## Discussion

In this systematic review, a global dataset, spanning 7 years, was systematically collated to investigate the epidemiological profile of AIV and its’ subtypes. Our findings suggest that AIV still exists and expands. H5N1 remains the most dominant AIV subtype. Moreover, regarding the geographical extent of outbreaks, Asia is the prevalent continent. China, Viet Nam, India, Taiwan, Israel, Japan and South Korea have reported some of the largest outbreaks during 2010–2016. This can be attributed to the enhanced laboratory and clinically programmes implemented in all these countries over the last years or to the fact that these countries dispose a unique ecosystem with numerous lakes, ponds, creeks and rivers, constituting wintering areas for migratory birds. Also, Africa (Egypt, Nigeria, Ghana, Cameroon, Togo, South Africa, Tunisia, Cote d’Ivoire, Burkina Faso, Libya) reported a great proportion of cases. The expand of the virus in Europe, especially the spread of HPAI H5N8 in Germany, strengthens the close relation between the spread of the virus via wild bird migration and their habitats. Our results, combined with the data from all the past 15 years, show that influenza activity may change from year to year and season to season. Although the drivers/reasons are not fully understood, a correlation with certain climatic conditions is proposed [27, 28]. Our results, also, demonstrate a difference in reported cases based on serologically tested samples when compared with those confirmed by molecular testing. This difference seems to be expected, as it is due to cases serologically identified, but not confirmed and characterised molecularly. This could be both attributed to possible differences in validity (sensitivity and specificity) of all methods of testing and to the fact that none of the diagnostic tests are validated for all species or specimen types [29].

Concerning the age factor, we should consider that in commercial species, in particular broilers, the rearing period is highly standardised worldwide. In some cases, age was specified as a week before reaching slaughter or at week 4 of the rearing period. In all of these cases, we considered that information on “age” was not given. Also, there were outbreaks referring to a number of flocks within a region, where it was likely that several ages were involved, i.e. one outbreak involving 20 farms in the same region each with birds of a certain age. This should be also taken into account regarding the validity of AIV distribution by age.

Overall, our evidence robustly reports that AIV exists and is disseminated worldwide. Our systematic review has several limitations that could be divided into two separate categories: firstly, there are limitations of the methodology of the review per se and secondly, limitations related to the variable quality of the source-articles. The identification of numerous references obtained through the lists of the studies distinguished by search engines, i.e. 8% of all citations included, indicates that the search strategies and the eligibility criteria sets may have been very specific and restrictive. Concerning the source limitations, the population of the avian species, the surveillance and diagnostic procedures and the recording system applied in each country, as well as the state of the country, are not the same among the studies evaluated. Analyses and data referring to cases that reported the application of vaccination programme could not be confirmed, as flock histories (percentage of vaccination, doses of vaccination applied, etc.) are often not available [30]. For example, vaccine composition in Viet Nam generates an immune response that cannot be distinguished from natural infection [31]. It has been proven to be difficult to achieve the ideal balance between sensitivity and specificity, as time, keywords and resource constraints are set in this paper. Publication bias might account for this observed effect. The keywords set in our search strategy were “avian influenza” and “avian influenza virus” and “avian flu” and “outbreak” and concerned the article title, abstract or keywords. Thus, papers missing to mention the above key terms in their titles, abstracts or keywords, albeit reporting them in their full text, may have also been missed. Another point that had to be clarified was whether AIV cases were related to human or avian species. A great number of studies which were identified were referring to human infections of AIV. We, therefore, believe and suggest that future reviews should follow a more comprehensive search strategy and approach. Furthermore, taking into account that none, except for 13 of the included studies, scored the maximum on the quality assessment; there is a concern for the methodological quality of this systematic review and the risk of bias of the included studies. This may be attributed to various factors, such as sample heterogeneity, variety of statistical analyses followed and others.

The cases identified in this systematic review are likely to be underestimates of the real incidence of AIV, due to the limiting timeline of 2010–2016 set by us and difficulties of precisely scrutinising and including data referring to years 2010–2016. There were outbreaks reporting massive numbers of cases between 2007 and 2012, which did not separate cases per year and were rejected since they did not allow us to “isolate” the number of cases concerning the timeline set by us (2010–2016). Moreover, it has been proven very difficult to make comparisons among the outbreaks included, as there were vast differentiations in avian influenza subtypes, environmental and geospatial conditions and key characteristics of affected populations. Wherever mentioned, there was, also, a solid variation in case definitions among the outbreaks identified in this systematic review, e.g. an outbreak within a flock in which a number of infected birds is reported, an outbreak in a region involving flocks and with no information about number of cases in each flock (i.e. the unit is the flock), an outbreak that in fact is not an outbreak, but an endemic situation that just happens to be reported. The issue of the definition of the outbreak, namely the level at which the outbreak is defined and the extent to which it is reported as an outbreak, should be considered carefully. Hence, the calculation of the attack rate is likely to vary by study due to methodological differences.

This review, also, suffers from a lack of details provided by the primary papers. For example, it was very difficult to assess the degree of association between the vicinity of water mentioned and AIV cases, because of the limited amount of relevant information that was available, particularly in the ProMED mail reports.

Deficiencies emerging from our review draw attention to key areas that future outbreak reports should try to address. Future studies in this area should aim to record the presence of wild birds that are thought to be involved, while effort should, also, focus on possible routes of transmission. This could allow a greater number of studies to be assessed and included in reviews like this one. Generally, anything that is considered to be implicated (weather conditions, water collections, etc.) should be clearly stated. The case definition should be clearly remarked, as well. It is of great importance to highlight the contingent role of such factors and to encourage researchers to explicitly investigate whether such factors occurred prior to the outbreak or after the outbreak.

The global distribution of avian influenza outbreaks or cases as mentioned in the scientific literature is very likely to be subject to considerable publication bias. A greater proportion of these outbreaks identified through ProMED mail was in Asia compared to those reported in peer-reviewed journals. Outbreaks in countries that apply preventive measures and follow surveillance and vaccination programmes are often thought to be easily controlled [32]. The scientific literature is, also, likely to originate from countries with greater academic, financial and surveillance capacity. Therefore, the number and type of studies published may not be proportionate to the extent and consequences of an outbreak. ProMED mail is a passive reporting tool, and this must be taken into account, as discrepancies are really possible to appear. For instance, outbreaks which occurred in countries of international interest, with potential public health risks (i.e. China) and minor commercial effects, were more likely to be reported [33].

The scientific literature confirms that all haemagglutinin and neuraminidase influenza A subtypes of possible combinations have been identified from avian species and affect all type of domestic or captive birds, worldwide [34]. Nevertheless, our results demonstrate a difference in H5 and H7 rates, which are regarded as common virulent subtypes that are characterised as highly pathogenic avian influenza viruses (HPAI) [34]. These subtypes, particularly H5N1, H5N8, H5, H5N6, mixed, H5N2 and H7N9, were also found to be more dominant in the wild bird cases of this systematic review. Moreover, our findings agree with those of Bui et al., according to whom H7N9 is not highly pathogenic in wild birds, whereas we also concluded that H5N1 cases remain consistent worldwide, via wild bird migration and poultry trade activities [35]. Bui et al. found that H7N9 in wild birds occurred largely in a great number of contiguous provinces of China, and our results support this evidence, as all H7N9 cases of our systematic review took place in China [35]. Bui et al. also assume that species with affinity to water collections and coastal places are considered to be primary reservoirs of AIVs. Cases of H5N1 affected a greater area of China compared to those of H7N9 infection [35]. Van Kerkhove et al. recognise endemicity of H5N1 subtype in several parts of Asia and Egypt [36]. Wan et al. concluded that, indeed, China is an AIV epicentre, as minimum two large pandemics (1957 and 1968) originated from there, spreading across Asia, Africa and Europe [37].

## Conclusions

This systematic review suggests that AIV outbreaks do occur in both developed and developing countries, and this, per se, constitutes an important burden on public health. Further epidemiological studies may contribute to identify possible risk factors and understand the extent and routes of the spread of AIV. In terms of consistent reporting, proper case definitions (farm, flock, bird, etc.) should also be designated when outbreaks are reported. Systematic surveillance and prevention programmes shall continue to be enhanced in “risky” countries (e.g. China, Viet Nam), while auditing should be imposed in wet and live bird markets in order to ensure safe and appropriate animal practices.

## Additional file

Additional file 1: Table S1. Avian influenza birds affected per virus subtype, country, epidemiological unit and year. References for Table S1. (ZIP 230 kb)

## Abbreviations

AIV: Avian influenza virus
HA: Haemagglutinin
HPAI: Highly pathogenic avian influenza
IVPI: Intravenous pathogenicity index
LPAI: Low pathogenic avian influenza
NA: Neuraminidase
OIE: World Organisation for Animal Health
