# Supplementary material for: Global avian influenza outbreaks 2010–2016: a systematic review of their distribution, avian species and virus subtype
Source: Syst Rev. 2018 Jan 25;7:17. doi: 10.1186/s13643-018-0691-z (PMC5784696; doi:10.1186/s13643-018-0691-z)
Supplement: Additional file 1: Table S1. — Avian influenza birds affected per virus subtype, country, epidemiological unit and year. References for Table S1. (ZIP 230 kb) [file 13643_2018_691_MOESM1_ESM.zip › Additional file 1.pdf]

Additional file 1

Table S1 references

(Marchenko et al., 2011)(Z. Fan et al., 2015)(Gugong et al., 2012)(Śmietanka et al., 2014)(Madsen, Zimmermann, Timmons, & Tablante, 2013)(Coker, Meseko, Odaibo, & Olaleye, 2014)(J.a Han et al., 2013)(Hu et al., 2015)(Y. . Huang, Robertson, Ojkic, Whitney, & Lang, 2014)(Tosh et al., 2011)(Creanga et al., 2013)(Ge et al., 2014)(Ali et al., 2015)(M. J. Slomka et al., 2012)(Yu et al., 2015)(Parker et al., 2012)(Hiono et al., 2015)(H Wu et al., 2015)(Nidom et al., 2012)(Haibo Wu, Peng, et al., 2015)(Hai-bo Wu et al., 2012)(Vuong N Bui et al., 2013)(Lee et al., 2015)(Yoon et al., 2015)(Hye-Ryoung Kim et al., 2012)(Bi et al., 2015)(Ku et al., 2014)(Jairak et al., 2016)(P. Y. Huang et al., 2016)(Núñez et al., 2016)(Conraths et al., 2016)(Cuong et al., 2016)(M. Fan et al., 2014)(Amen et al., 2015)(Arzey et al., 2012)(Haibo Wu, Lu, et al., 2015)(Shi et al., 2013) (Dubey et al., 2012)(Okuya et al., 2015)(Shen et al., 2015)(El-Zoghby, Arafa, Kilany, et al., 2012)(El-Zoghby, Arafa, Hassan, et al., 2012)(Haibo Wu et al., 2014)(Zhu et al., 2012)(Bahari, Pourbakhsh, Shoushtari, & Bahmaninejad, 2015)(S.-H. Kim et al., 2015)(Chang et al., 2014)(Khatun et al., 2013)(Oluwayelu, Aiki-Raji, Adigun, Olofintuyi, & Adebiyi, 2015)(Hai-bo et al., 2012)(Kirunda et al., 2014)(Jhung & Nelson, 2015)(Sohaib et al., 2010)(Z. Fan et al., 2015)(H.-R. Kim et al., 2015)(Hagag et al., 2015)(Mansour, ElBakrey, Ali, Knudsen, & Eid, 2014)(Abdo et al., 2014)(Ip et al., 2015) (Kang et al., 2015)(Sheta et al., 2014)(Vuong Nghia Bui et al., 2014)(Beaudoin et al., 2014)(Shin et al., 2015)(Pu et al., 2015)(Jiankang Han et al., 2014)(Okamatsu et al., 2013)(Osmani, Ward, Giasuddin, Islam, & Kalam, 2014)(J.a Han et al., 2014)(Lindh et al., 2014)(Khan et al., 2014)(Haider et al., 2015)(M. J. . Slomka et al., 2013)

References

1. Abdo, W. . d, Haridy, M. . b, Katou, Y. ., Goto, M. ., Mizoguchi, T. ., Sakoda, Y. ., … Yanai, T. . (2014). Pathological and immunohistochemical findings of natural highly pathogenic avian influenza infection in tufted ducks during 2010–2011 outbreaks in Japan. *Journal of Veterinary Medical Science*, *76*(9), 1285–1290. http://doi.org/10.1292/jvms.14-0084
2. Abolnik C. A current review of avian influenza in pigeons and doves (Columbidae). Vet Microbiol. Elsevier B.V.; 2014;170(3–4):181–96.
3. Alexander DJ. Report on avian influenza in the Eastern Hemisphere during 1997-2002. Avian Dis [Internet]. 2003;47(3 Suppl):792–7. Available from: http://www.ncbi.nlm.nih.gov/pubmed/14575066
4. Alexander DJ. A review of avian influenza in different bird species. Vet Microbiol. 2000;74(1–2):3–13.
5. Ali, A., Elmowalid, G., Abdel-Glil, M., Sharafeldin, T., Abdallah, F., Mansour, S., … Abdelmoneim, M. (2015). Etiology and pathology of epidemic outbreaks of avian influenza H5N1 infection in Egyptian chicken farms. *Polish Journal of Veterinary Sciences*, *18*(4), 779–786. http://doi.org/10.1515/pjvs-2015-0101
6. Amen, O. . b, Vemula, S. V. ., Zhao, J. ., Ibrahim, R. ., Hussein, A. ., Hewlett, I. K. ., … Mittal, S. K. . (2015). Identification and characterization of a highly pathogenic H5N1 avian influenza A virus during an outbreak in vaccinated chickens in Egypt. *Virus Research*, *210*, 337–343. http://doi.org/10.1016/j.virusres.2015.09.004
7. Arzey, G. G. ., Kirkland, P. D. ., Arzey, K. E. ., Frost, M. ., Maywood, P. .,

Conaty, S. ., … Selleck, P. . (2012). Influenza virus a (H10N7) in chickens and poultry abattoir workers, Australia. *Emerging Infectious Diseases*, *18*(5), 814–816. http://doi.org/10.3201/eid1805.111852

8. Bahari, P., Pourbakhsh, S. A., Shoushtari, H., & Bahmaninejad, M. A. (2015). Molecular characterization of H9N2 avian influenza viruses isolated from vaccinated broiler chickens in northeast Iran. *Tropical Animal Health and Production*, *47*(6), 1195–201. http://doi.org/10.1007/s11250-015-0848-x
9. Beaudoin, A. L. ., Kitikoon, P. ., Schreiner, P. J. ., Singer, R. S. ., Sasipreeyajan, J. ., Amonsin, A. ., … Bender, J. B. . (2014). Risk factors for exposure to influenza a viruses, including subtype H5 viruses, in Thai free-grazing ducks. *Transboundary and Emerging Diseases*, *61*(4), 362–374. http://doi.org/10.1111/tbed.12043
10. Bellou M, Kokkinos P, Vantarakis A. Shellfish-Borne Viral Outbreaks: A Systematic Review. Food Env Virol. 2013;5(1):13–23.
11. Bi, Y. . b f, Zhang, Z. ., Liu, W. . f, Yin, Y. ., Hong, J. ., Li, X. ., … Lei, F. . f. (2015). Highly Pathogenic Avian Influenza A(H5N1) Virus Struck Migratory Birds in China in 2015. *Scientific Reports*, *5*. http://doi.org/10.1038/srep12986
12. Brown JD, Goekjian G, Poulson R, Valeika S, Stallknecht DE. Avian influenza virus in water: Infectivity is dependent on pH, salinity and temperature. Vet Microbiol. 2009;136(1–2):20–6.
13. Bui C, Bethmont A, Chughtai AA, Gardner L, Sarkar S, Hassan S, et al. A Systematic Review of the Comparative Epidemiology of Avian and Human Influenza A H5N1 and H7N9 - Lessons and Unanswered Questions. Transbound Emerg Dis. 2015;1–19.
14. Bui, V. N., Ogawa, H., Ngo, L. H., Baatartsogt, T., Abao, L. N. B., Tamaki,

S., … Imai, K. (2013). H5N1 highly pathogenic avian influenza virus isolated from conjunctiva of a whooper swan with neurological signs. *Archives of Virology*, *158*(2), 451–5. http://doi.org/10.1007/s00705-012-1502-9

15. Bui, V. N., Ogawa, H., Trinh, D. Q., Nguyen, T. H. T., Pham, N. T., Truong, D. A., … Nguyen, K. V. (2014). Genetic characterization of an H5N1 avian influenza virus from a vaccinated duck flock in Vietnam. *Virus Genes*, *49*(2), 278–85. http://doi.org/10.1007/s11262-014-1089-1
16. Chang, H., Dai, F., Liu, Z., Yuan, F., Zhao, S., Xiang, X., … Duan, G. (2014). Seroprevalence survey of avian influenza A (H5) in wild migratory birds in Yunnan Province, Southwestern China. *Virology Journal*, *11*, 18. http://doi.org/10.1186/1743-422X-11-18
17. Coker, T., Meseko, C., Odaibo, G., & Olaleye, D. (2014). Circulation of the low pathogenic avian influenza subtype H5N2 virus in ducks at a live bird market in Ibadan, Nigeria. *Infectious Diseases of Poverty*, *3*(1), 38. http://doi.org/10.1186/2049-9957-3-38
18. Conraths, F. J. ., Sauter-Louis, C. ., Globig, A. ., Dietze, K. ., Pannwitz, G. ., Albrecht, K. ., … Homeier-Bachmann, T. . (2016). Highly Pathogenic Avian Influenza H5N8 in Germany: Outbreak Investigations. *Transboundary and Emerging Diseases*, *63*(1), 10–13. http://doi.org/10.1111/tbed.12443
19. Creanga, A. . b, Thi Nguyen, D. . d e, Gerloff, N. ., Thi Do, H. ., Balish, A. ., Dang Nguyen, H. ., … Nguyen, T. . f. (2013). Emergence of multiple clade 2.3.2.1 influenza A (H5N1) virus subgroups in Vietnam and detection of novel reassortants. *Virology*, *444*(1–2), 12–20. http://doi.org/10.1016/j.virol.2013.06.005
20. Cuong, N. V, Truc, V. N. T., Nhung, N. T., Thanh, T. T., Chieu, T. T. B.,

Hieu, T. Q., … Hoa, N. T. (2016). Highly Pathogenic Avian Influenza Virus A/H5N1 Infection in Vaccinated Meat Duck Flocks in the Mekong Delta of Vietnam. *Transboundary and Emerging Diseases*. http://doi.org/10.1111/tbed.12470

21. Dean AG. Population-Based Spot Maps : An Epidemiologic Technique. 1976;66(10):988–9.
22. Disease outbreaks. World Health Organisation. 2016.
23. Dubey, S. C. ., Dahal, N. ., Nagarajan, S. ., Tosh, C. ., Murugkar, H. V. ., Rinzin, K. ., … Krishna, L. . (2012). Isolation and characterization of influenza A virus (subtype H5N1) that caused the first highly pathogenic avian influenza outbreak in chicken in Bhutan. *Veterinary Microbiology*, *155*(1), 100–105. http://doi.org/10.1016/j.vetmic.2011.08.002
24. Ellis TM, Leung CYHC, Chow MKW, Bissett L a, Wong W, Guan Y, et al. Vaccination of chickens against H5N1 avian influenza in the face of an outbreak interrupts virus transmission. Avian Pathol. 2004;33(4):405–12.
25. El-Zoghby, E. F., Arafa, A.-S., Kilany, W. H., Aly, M. M., Abdelwhab, E. M., & Hafez, H. M. (2012). Isolation of avian influenza H5N1 virus from vaccinated commercial layer flock in Egypt. *Virology Journal*, *9*(1), 294. http://doi.org/10.1186/1743-422X-9-294
26. El-Zoghby, E. F., Arafa, A. S., Hassan, M. K., Aly, M. M., Selim, A., Kilany, W. H., … Hafez, H. M. (2012). Isolation of H9N2 avian influenza virus from bobwhite quail (Colinus virginianus) in Egypt. *Archives of Virology*, *157*(6), 1167–1172. http://doi.org/10.1007/s00705-012-1269-z
27. Fan, M., Huang, B., Wang, A., Deng, L., Wu, D., Lu, X., … Xiang, N. (2014). Human influenza A(H7N9) virus infection associated with poultry farm,

Northeastern China. *Emerging Infectious Diseases*, *20*(11), 1902–1905. http://doi.org/10.3201/eid2011.140608

28. Fan, Z., Ci, Y., Liu, L., Ma, Y., Jia, Y., Wang, D., … Chen, H. (2015). Phylogenetic and pathogenic analyses of three H5N1 avian influenza viruses (clade 2.3.2.1) isolated from wild birds in Northeast China. *Infection, Genetics and Evolution*, *29*, 138–145. http://doi.org/10.1016/j.meegid.2014.11.020
29. Fiebig L, Soyka J, Buda S, Buchholz U, Dehnert M, Haas W. Avian influenza A(H5N1) in humans: New insights from a line list of World Health Organization confirmed cases, September 2006 to August 2010. Eurosurveillance [Internet]. 2011;16(32). Available from: http://www.scopus.com/inward/record.url?eid=2-s2.0-80052058552&partnerID=40&md5=e7f7973f260a2ef5f6f36b28ff861f77
30. Ge, F.-F., Ju, H.-B., Yang, D.-Q., Liu, J., Wang, J., Lu, J., … Zhou, J.-P. (2014). Epidemiological situation and genetic analysis of H7N9 influenza viruses in Shanghai in 2013. *Archives of Virology*, *159*(11), 3029–41. http://doi.org/10.1007/s00705-014-2177-1
31. Gugong, V. T. ., Ajogi, I. ., Juniadu, K. ., Okolocha, E. C. ., Ngbede, E. O. ., Hambolu, S. E. ., & Maurice, N. A. . (2012). Avian influenza (H5 subtype) antibodies in village chickens in four local government areas of Kaduna state, Nigeria. *Veterinary World*, *5*(12), 713–717. http://doi.org/10.5455/vetworld.2012.713-717
32. Hagag, I. T. . b, Mansour, S. M. G. ., Zhang, Z. ., Ali, A. A. H. ., Ismaiel, E.-B. M. ., Salama, A. A. ., … Xing, Z. . d. (2015). Pathogenicity of highly pathogenic avian influenza virus H5N1 in naturally infected poultry in Egypt. *PLoS ONE*, *10*(5). http://doi.org/10.1371/journal.pone.0120061

33. Hai-bo, W., Ru-feng, L., En-kang, W., Jin-biao, Y., Yi-ting, W., Qiao-gang, W., … Chao-tan, G. (2012). Sequence and phylogenetic analysis of H7N3 avian influenza viruses isolated from poultry in China in 2011. *Archives of Virology*, *157*(10), 2017–2021. http://doi.org/10.1007/s00705-012-1370-3
34. Haider, N. . b, Sturm-Ramirez, K. . c, Khan, S. U. . d, Rahman, M. Z. ., Sarkar, S. ., Poh, M. K. ., … Zeidner, N. . b. (2015). Unusually high mortality in waterfowl caused by highly pathogenic avian influenza A(H5N1) in Bangladesh. *Transboundary and Emerging Diseases*. http://doi.org/10.1111/tbed.12354
35. Han, J. ., Niu, F. ., Jin, M. ., Wang, L. ., Liu, J. ., Zhang, P. ., … Lan, K. . (2013). Clinical presentation and sequence analyses of HA and NA antigens of the novel H7N9 viruses. *Emerging Microbes and Infections*, *2*. http://doi.org/10.1038/emi.2013.28
36. Han, J. ., Wang, L. ., Liu, J. ., Jin, M. ., Hao, F. ., Zhang, P. ., … Zhang, C. . (2014). Cocirculation of three hemagglutinin and two neuraminidase subtypes of avian influenza viruses in Huzhou, China, April 2013: Implication for the origin of the novel H7N9 Virus. *Journal of Virology*, *88*(11), 6506–6511. http://doi.org/10.1128/JVI.03319-13
37. Han, J., Liu, J., Wang, L., Zhang, P., Liu, G., Lan, K., & Zhang, C. (2014). Persistent detection of avian influenza A/H7N9 virus among poultry in Huzhou City, China, in the summer of 2013. *International Journal of Infectious Diseases*, *26*, 72–75. http://doi.org/10.1016/j.ijid.2014.01.020
38. Hiono, T., Ohkawara, A., Ogasawara, K., Okamatsu, M., Tamura, T., Chu, D.-H., … Kida, H. (2015). Genetic and antigenic characterization of H5 and H7 influenza viruses isolated from migratory water birds in Hokkaido, Japan and

Mongolia from 2010 to 2014. *Virus Genes*, *51*(1), 57–68. http://doi.org/10.1007/s11262-015-1214-9

39. Hu, M., Li, X., Ni, X., Wu, J., Gao, R., Xia, W., … Chen, H. (2015). Coexistence of avian influenza virus H10 and H9 subtypes among chickens in live poultrymarkets during an outbreak of infection with a novel H10N8 virus in humans in Nanchang, China. *Japanese Journal of Infectious Diseases*, *68*(5), 364–369. http://doi.org/10.7883/yoken.JJID.2014.377
40. Huang, P. Y., Lee, C. C. D., Yip, C. H., Cheung, C. L., Yu, G., Lam, T. T. Y., … Guan, Y. (2016). Genetic characterization of highly pathogenic H5 influenza viruses from poultry in Taiwan, 2015. *Infection, Genetics and Evolution*, *38*, 96–100. http://doi.org/10.1016/j.meegid.2015.12.006
41. Huang, Y. ., Robertson, G. J. ., Ojkic, D. ., Whitney, H. ., & Lang, A. S. . (2014). Diverse inter-continental and host lineage reassortant avian influenza A viruses in pelagic seabirds. *Infection, Genetics and Evolution*, *22*, 103–111. http://doi.org/10.1016/j.meegid.2014.01.014
42. Ip, H. S., Torchetti, M. K., Crespo, R., Kohrs, P., DeBruyn, P., Mansfield, K. G., … Sleeman, J. M. (2015). Novel Eurasian highly pathogenic avian influenza A H5 viruses in wild birds, Washington, USA, 2014. *Emerging Infectious Diseases*, *21*(5), 886–890. http://doi.org/10.3201/eid2105.142020
43. Jairak, W., Boonyapisitsopa, S., Chaiyawong, S., Nonthabenjawan, N., Tangwangvivat, R., Bunpapong, N., & Amonsin, A. (2016). Genetic characterization of influenza A (H7N6) virus isolated from a live-bird market in Thailand. *Archives of Virology*, *161*(5), 1315–1322. http://doi.org/10.1007/s00705-016-2759-1
44. James C. Control of communicable disease manual. Washington DC:

American Public Health Association; 2000.

45. Jhung, M. A. ., & Nelson, D. I. . (2015). Outbreaks of avian influenza a (H5N2), (H5N8), and (H5N1) among birds — United States, December 2014–January 2015. *Morbidity and Mortality Weekly Report*, *64*(4), 111. Retrieved from http://www.scopus.com/inward/record.url?eid=2-s2.0-84922449372&partnerID=40&md5=b0cfbebdc29f122c79747e93f0116b29
46. Kang, H.-M., Lee, E.-K., Song, B.-M., Jeong, J., Choi, J.-G., Jeong, J., … Lee, Y.-J. (2015). Novel reassortant influenza a(H5N8) viruses among inoculated domestic and wild ducks, South Korea, 2014. *Emerging Infectious Diseases*, *21*(2), 298–304. http://doi.org/10.3201/eid2102.141268
47. Khan, S. U., Berman, L. S., Haider, N., Gerloff, N., Rahman, M. Z., Shu, B., … Mikolon, A. B. (2014). Investigating a crow die-off in January-February 2011 during the introduction of a new clade of highly pathogenic avian influenza virus H5N1 into Bangladesh. *Archives of Virology*, *159*(3), 509–518. http://doi.org/10.1007/s00705-013-1842-0
48. Khatun, A. A. ., Giasuddin, M. . M., Islam, K. M. K. M. ., Khanom, S. . S., Samad, M. A. . M. A., Islam, M. R. . M. R., … Rahman, M. M. . d M. M. (2013). Surveillance of avian influenza virus type A in semi-scavenging ducks in Bangladesh. *BMC Veterinary Research*, *9*, 196. http://doi.org/10.1186/1746-6148-9-196
49. Kim, H.-R., Kwon, Y.-K., Jang, I., Lee, Y.-J., Kang, H.-M., Lee, E.-K., … Bae, Y.-C. (2015). Pathologic changes in wild birds infected with highly pathogenic avian influenza A(H5N8) viruses, South Korea, 2014. *Emerging Infectious Diseases*, *21*(5), 775–780. http://doi.org/10.3201/eid2105.141967
50. Kim, H.-R., Lee, Y.-J., Park, C.-K., Oem, J.-K., Lee, O.-S., Kang, H.-M., …

Bae, Y.-C. (2012). Highly pathogenic avian influenza (H5N1) outbreaks in wild birds and poultry, South Korea. *Emerging Infectious Diseases*, *18*(3), 480–3. http://doi.org/10.3201/eid1803.111490

51. Kim, S.-H., Hur, M., Suh, J.-H., Woo, C., Wang, S.-J., Park, E.-R., … Song, C.-S. (2015). Molecular characterization of highly pathogenic avian influenza H5N8 viruses isolated from Baikal teals found dead during a 2014 outbreak in Korea. *Journal of Veterinary Science*.
52. Kirunda, H., Erima, B., Tumushabe, A., Kiconco, J., Tugume, T., Mulei, S., … Wabwire-Mangen, F. (2014). Prevalence of influenza A viruses in livestock and free-living waterfowl in Uganda. *BMC Veterinary Research*, *10*, 50. http://doi.org/10.1186/1746-6148-10-50
53. Ku, K. B., Park, E. H., Yum, J., Kim, J. A., Oh, S. K., & Seo, S. H. (2014). Highly pathogenic avian influenza A(H5N8) virus from Waterfowl, South Korea, 2014. *Emerging Infectious Diseases*, *20*(9), 1587–1588. http://doi.org/10.3201/eid2009.140390
54. Lai S, Qin Y, Cowling BJ, Ren X, Wardrop NA, Gilbert M, et al. Global epidemiology of avian influenza A H5N1 virus infection in humans, 1997-2015: A systematic review of individual case data. Lancet Infect Dis. 2016;16(7):e108–18.
55. Lee, E.-K., Kang, H.-M., Kim, K.-I., Choi, J.-G., To, T. L., Nguyen, T. D., … Kim, J.-H. (2015). Genetic evolution of H5 highly pathogenic avian influenza virus in domestic poultry in Vietnam between 2011 and 2013. *Poultry Science*, *94*(4), 650–61. http://doi.org/10.3382/ps/pev036
56. Liberati A, Altman DG, Tetzlaff J, Mulrow C, Ioannidis JP a, Clarke M, et al. The PRISMA Statement for Reporting Systematic Reviews and Meta-

Analyses of Studies That Evaluate Health Care Interventions : explanation and elaboration. Ann Intern Med. 2009;151(4):W65–94.

57. Lindh, E. ., Ek-Kommonen, C. ., Väänänen, V.-M. ., Vaheri, A. . d, Vapalahti, O. . d e, & Huovilainen, A. . (2014). Molecular epidemiology of H9N2 influenza viruses in Northern Europe. *Veterinary Microbiology*, *172*(3–4), 548–554. http://doi.org/10.1016/j.vetmic.2014.06.020
58. Long NT, Thanh TT, van Doorn HR, Vu PP, Dung PT, Dung TTK, et al. Recent avian influenza virus A/H5N1 evolution in vaccinated and unvaccinated poultry from farms in Southern Vietnam, January-March 2010. Transbound Emerg Dis. 2011;58(6):537–43.
59. Madsen, J. M. ., Zimmermann, N. G. ., Timmons, J. ., & Tablante, N. L. . (2013). Avian Influenza Seroprevalence and Biosecurity Risk Factors in Maryland Backyard Poultry: A Cross-Sectional Study. *PLoS ONE*, *8*(2). http://doi.org/10.1371/journal.pone.0056851
60. Mansour, S. M. G., ElBakrey, R. M., Ali, H., Knudsen, D. E. B., & Eid, A. a. M. (2014). Natural infection with highly pathogenic avian influenza virus H5N1 in domestic pigeons (Columba livia) in Egypt. *Avian Pathology*, *43*(4), 319–324. http://doi.org/10.1080/03079457.2014.926002
61. Marchenko, V. I. Y., Sharshov, K. A., Silko, N. I. Y., Susloparov, I. M., Durymanov, A. G., ZaĭkovskaIa, A. V, … Shestopalov, A. M. (2011). [Characterization of the H5N1 influenza virus isolated during an outbreak among wild birds in Russia (Tuva Republic) in 2010]. *Molekuliarnaia Genetika, Mikrobiologiia I Virusologiia*, *26*(4), 36–40. http://doi.org/10.3103/S0891416811040057

62. Nidom, C. A., Yamada, S., Nidom, R. V, Rahmawati, K., Alamudi, M. Y., Kholik, … Kawaoka, Y. (2012). Genetic characterization of H5N1 influenza viruses isolated from chickens in Indonesia in 2010. *Virus Genes*, *44*(3), 459–65. http://doi.org/10.1007/s11262-012-0722-0

63. Núñez, A. ., Brookes, S. M. . M., Reid, S. M. . M., Garcia-Rueda, C. ., Hicks, D. J. . J., Seekings, J. M. . M., … Brown, I. H. . H. (2016). Highly Pathogenic Avian Influenza H5N8 Clade 2.3.4.4 Virus: Equivocal Pathogenicity and Implications for Surveillance Following Natural Infection in Breeder Ducks in the United Kingdom. *Transboundary and Emerging Diseases*, *63*(1), 5–9. http://doi.org/10.1111/tbed.12442

64. Okamatsu, M., Nishi, T., Nomura, N., Yamamoto, N., Sakoda, Y., Sakurai, K., … Kida, H. (2013). The genetic and antigenic diversity of avian influenza viruses isolated from domestic ducks, muscovy ducks, and chickens in northern and southern Vietnam, 2010-2012. *Virus Genes*, *47*(2), 317–29. http://doi.org/10.1007/s11262-013-0954-7

65. Okuya, K., Kawabata, T., Nagano, K., Tsukiyama-Kohara, K., Kusumoto, I., Takase, K., & Ozawa, M. (2015). Isolation and characterization of influenza A viruses from environmental water at an overwintering site of migratory birds in Japan. *Archives of Virology*, *160*(12), 3037–52. http://doi.org/10.1007/s00705-015-2610-0

66. Oluwayelu, D. O., Aiki-Raji, C. O., Adigun, O. T., Olofintuyi, O. K., & Adebiyi, A. I. (2015). Serological Survey for Avian Influenza in Turkeys in Three States of Southwest Nigeria. *Influenza Research and Treatment*, *2015*, 787890. http://doi.org/10.1155/2015/787890

67. Osmani, M. G. ., Ward, M. P. ., Giasuddin, M. ., Islam, M. R. ., & Kalam, A. .

(2014). The spread of highly pathogenic avian influenza (subtype H5N1) clades in Bangladesh, 2010 and 2011. *Preventive Veterinary Medicine*, *114*(1), 21–27. http://doi.org/10.1016/j.prevetmed.2014.01.010

68. Parker, C. D., Reid, S. M., Ball, A., Cox, W. J., Essen, S. C., Hanna, A., … Brown, I. H. (2012). First reported detection of a low pathogenicity avian influenza virus subtype H9 infection in domestic fowl in England. *The Veterinary Record*, *171*(15), 372. http://doi.org/10.1136/vr.100558
69. Phuong DQ. b c, Dung NT., Joørgensen PH., van Thanh D., Tung DD., Christensen JP. Virulence of H5N1 influenza virus in cattle egrets (Bubulcus IBIS). J Wildl Dis [Internet]. 2011;47(2):314–20. Available from: http://www.scopus.com/inward/record.url?eid=2-s2.0-79953665853&partnerID=40&md5=ec7d8875e58a98e40ed61e899fb4864e
70. Principles of Epidemiology in Public Health Practice, Third Edition An Introduction to Applied Epidemiology and Biostatistics [Internet]. Centres for Disease Control and Prevention. p. 2012. Available from: http://www.cdc.gov/ophss/csels/dsepd/ss1978/lesson1/section11.html
71. Pu, J., Wang, S., Yin, Y., Zhang, G., Carter, R. A., Wang, J., … Webster, R. G. (2015). Evolution of the H9N2 influenza genotype that facilitated the genesis of the novel H7N9 virus. *Proceedings of the National Academy of Sciences of the United States of America*, *112*(2), 548–553. http://doi.org/10.1073/pnas.1422456112
72. Shen, H.-Q. ., Yan, Z.-Q. ., Zeng, F.-G. ., Liao, C.-T. ., Zhou, Q.-F. ., Qin, J.-P. ., … Chen, F. . (2015). Isolation and phylogenetic analysis of hemagglutinin gene of H9N2 influenza viruses from chickens in South China from 2012 to 2013. *Journal of Veterinary Science*, *16*(3), 317–324.

http://doi.org/10.4142/jvs.2015.16.3.317

73. Sheta, B. M. . b, Fuller, T. L. ., Larison, B. ., Njabo, K. Y. ., Ahmed, A. S. ., Harrigan, R. ., … Smith, T. B. . (2014). Putative human and avian risk factors for avian influenza virus infections in backyard poultry in Egypt. *Veterinary Microbiology*, *168*(1), 208–213. http://doi.org/10.1016/j.vetmic.2013.11.010
74. Shi, J., Deng, G., Liu, P., Zhou, J., Guan, L., Li, W., … Chen, H. (2013). Isolation and characterization of H7N9 viruses from live poultry markets — Implication of the source of current H7N9 infection in humans. *Chinese Science Bulletin*, *58*(16), 1857–1863. http://doi.org/10.1007/s11434-013-5873-4
75. Shin, J.-H. . J.-H., Woo, C., Wang, S.-J. . S.-J., Jeong, J. J. ., An, I.-J. . I.-J., Hwang, J.-K. . J.-K., … Kim, S.-H. . S.-H. (2015). Prevalence of avian influenza virus in wild birds before and after the HPAI H5N8 outbreak in 2014 in South Korea. *Journal of Microbiology*, *53*(7), 475–80. http://doi.org/10.1007/s12275-015-5224-z
76. Slomka, M. J. ., Hanna, A. ., Mahmood, S. ., Govil, J. ., Krill, D. ., Manvell, R. J. ., … Brown, I. H. . (2013). Phylogenetic and molecular characteristics of Eurasian H9 avian influenza viruses and their detection by two different H9-specific RealTime reverse transcriptase polymerase chain reaction tests. *Veterinary Microbiology*, *162*(2–4), 530–542. http://doi.org/10.1016/j.vetmic.2012.11.013
77. Slomka, M. J., To, T. L., Tong, H. H., Coward, V. J., Mawhinney, I. C., Banks, J., & Brown, I. H. (2012). Evaluation of lateral flow devices for identification of infected poultry by testing swab and feather specimens during H5N1 highly pathogenic avian influenza outbreaks in Vietnam. *Influenza and*

Other Respiratory Viruses, *6*(5), 318–327. http://doi.org/10.1111/j.1750-2659.2011.00317.x

78. Śmietanka, K., Minta, Z., Świętoń, E., Olszewska, M., Jóźwiak, M., Domańska-Blicharz, K., … Pikuła, A. (2014). Avian influenza H9N2 subtype in Poland – characterization of the isolates and evidence of concomitant infections. *Avian Pathology*, *43*(5), 427–436. http://doi.org/10.1080/03079457.2014.952221
79. Sohaib, M., Siddique, M., Muhammad, K., Rabbani, M., Altaf, I., & Hanif, A. (2010). Prevalence of avian influenza virus (H5) in poultry layer flocks in and around Faisalabad, Punjab, Pakistan. *Pakistan Journal of Zoology*, *42*(3), 325–329.
80. Spackman E. A brief introduction to Avian Influenza Virus. In: Spackman E, editor. Animal Influenza Virus. Springer New York; 2014. p. 61–8.
81. Suarez DL, Das A, Ellis E. Review of rapid molecular diagnostic tools for avian influenza virus. Avian Dis. 2007;51(1 Suppl):201–8.
82. Tong S, Li Y, Rivailler P, Conrardy C, Castillo DAA, Chen L-M, et al. A distinct lineage of influenza A virus from bats. Proc Natl Acad Sci U S A. 2012;109(11):4269–74.
83. 7. Tong S, Zhu X, Li Y, Shi M, Zhang J, Bourgeois M, et al. New World Bats Harbor Diverse Influenza A Viruses. PLoS Pathog. 2013;9(10):e1003657.
84. Tosh, C., Murugkar, H. V, Nagarajan, S., Tripathi, S., Katare, M., Jain, R., … Dubey, S. C. (2011). Emergence of amantadine-resistant avian influenza H5N1 virus in India. *Virus Genes*, *42*(1), 10–5. http://doi.org/10.1007/s11262-010-0534-z

85. Van Kerkhove MD, Mumford E, Mounts AW, Bresee J, Ly S, Bridges CB, et al. Highly pathogenic avian influenza (H5N1): Pathways of exposure at the animal-human interface, a systematic review. PLoS One. 2011;6(1):1–8.
86. Wan XF. Lessons from Emergence of A/Goose/Guangdong/1996-Like H5N1 Highly Pathogenic Avian Influenza Viruses and Recent Influenza Surveillance Efforts in Southern China. Zoonoses Public Health. 2012;59(SUPPL.2):32–42.
87. Webster RG, Laver WG. Antigenic variation of influenza viruses. The Influenza Viruses and Influenza. New YorkAcademic Press; 1975. p. 270–314.
88. World Organisation For Animal Health. Manual of Diagnostic Tests and Vaccines for Terrestrial Animals. OIE. 2015.
89. Wu, H., Guo, C., Lu, R., Xu, L., Wo, E., You, J., … Wu, N. (2012). Genetic characterization of subtype H1 avian influenza viruses isolated from live poultry markets in Zhejiang Province, China, in 2011. *Virus Genes*, *44*(3), 441–9. http://doi.org/10.1007/s11262-012-0716-y
90. Wu, H., Lu, R., Wu, X., Peng, X., Xu, L., Cheng, L., … Wu, N. (2015). Isolation and characterization of a novel H10N2 avian influenza virus from a domestic duck in Eastern China. *Infection, Genetics and Evolution*, *29*, 1–5. http://doi.org/10.1016/j.meegid.2014.10.029
91. Wu, H., Peng, X., Peng, X., Cheng, L., Lu, X., Jin, C., … Wu, N. (2015). Genetic and molecular characterization of H9N2 and H5 avian influenza viruses from live poultry markets in Zhejiang Province, eastern China. *Scientific Reports*, *5*. http://doi.org/10.1038/srep17508
92. Wu, H., Peng, X., Peng, X., Cheng, L., Lu, X., Jin, C., … Wu, N. (2015). Genetic characterization of natural reassortant H4 subtype avian influenza

viruses isolated from domestic ducks in Zhejiang province in China from 2013 to 2014. *Virus Genes*, *51*(3), 347–55. http://doi.org/10.1007/s11262-015-1245-2

93. Wu, H., Wu, N., Peng, X., Jin, C., Lu, X., Cheng, L., … Li, L. (2014). Molecular characterization and phylogenetic analysis of H3 subtype avian influenza viruses isolated from domestic ducks in Zhejiang Province in China. *Virus Genes*, *49*(1), 80–8. http://doi.org/10.1007/s11262-014-1065-9
94. Yoon, H., Moon, O.-K., Jeong, W., Choi, J., Kang, Y.-M., Ahn, H.-Y., … Joo, Y.-S. (2015). H5N8 highly pathogenic avian influenza in the Republic of Korea: Epidemiology during the first wave, from January through July 2014. *Osong Public Health and Research Perspectives*, *6*(2), 106–111. http://doi.org/10.1016/j.phrp.2015.01.005
95. Yu, Z. . b g, Gao, X. ., Wang, T. . g, Li, Y. ., Li, Y. ., Xu, Y. ., … Gao, Y. . g h. (2015). Fatal H5N6 avian influenza virus infection in a domestic cat and wild birds in china. *Scientific Reports*, *5*. http://doi.org/10.1038/srep10704
96. Zhu, N., Zhao, J., Li, Y., Ding, C., Xia, H., Tang, S., … Li, T. (2012). Molecular characterization of H3N2 and H4N6 subtypes avian influenza viruses isolated from mallards in Poyang Lake, China in 2010. *Chinese Science Bulletin*, *57*(27), 3586–3594. http://doi.org/10.1007/s11434-012-5312-y
